# Bacteriophage treatment of carbapenemase-producing *Klebsiella pneumoniae* in a multispecies biofilm: a potential biocontrol strategy for healthcare facilities

**DOI:** 10.3934/microbiol.2020003

**Published:** 2020-02-26

**Authors:** Ariel J. Santiago, Maria L. Burgos-Garay, Leila Kartforosh, Mustafa Mazher, Rodney M. Donlan

**Affiliations:** Clinical and Environmental Microbiology Branch, Division of Healthcare Quality Promotion, Centers for Disease Control and Prevention, Atlanta, GA, USA

**Keywords:** biofilms, bacteriophage, carbapenemase-producing *Klebsiella pneumoniae*, healthcare-associated infections

## Abstract

The p-traps of hospital handwashing sinks represent a potential reservoir for antimicrobial-resistant organisms of major public health concern, such as carbapenemase-producing KPC+ *Klebsiella pneumoniae* (CPKP). Bacteriophages have reemerged as potential biocontrol agents, particularly against biofilm-associated, drug-resistant microorganisms. The primary objective of our study was to formulate a phage cocktail capable of targeting a CPKP strain (CAV1016) at different stages of colonization within polymicrobial drinking water biofilms using a CDC biofilm reactor (CBR) p-trap model. A cocktail of four CAV1016 phages, all exhibiting depolymerase activity, were isolated from untreated wastewater using standard methods. Biofilms containing *Pseudomonas aeruginosa*, *Micrococcus luteus*, *Stenotrophomonas maltophilia*, *Elizabethkingia anophelis*, *Cupriavidus metallidurans*, and *Methylobacterium fujisawaense* were established in the CBR p-trap model for a period of 28 d. Subsequently, CAV1016 was inoculated into the p-trap model and monitored over a period of 21 d. Biofilms were treated for 2 h at either 25 °C or 37 °C with the phage cocktail (10^9^ PFU/ml) at 7, 14, and 21 d post-inoculation. The effect of phage treatment on the viability of biofilm-associated CAV1016 was determined by plate count on m-Endo LES agar. Biofilm heterotrophic plate counts (HPC) were determined using R2A agar. Phage titers were determined by plaque assay. Phage treatment reduced biofilm-associated CAV1016 viability by 1 log_10_ CFU/cm^2^ (p < 0.05) at 7 and 14 d (37 °C) and 1.4 log_10_ and 1.6 log_10_ CFU/cm^2^ (p < 0.05) at 7 and 14 d, respectively (25 °C). No significant reduction was observed at 21 d post-inoculation. Phage treatment had no significant effect on the biofilm HPCs (p > 0.05) at any time point or temperature. Supplementation with a non-ionic surfactant appears to enhance phage association within biofilms. The results of this study suggest the potential of phages to control CPKP and other carbapenemase-producing organisms associated with microbial biofilms in the healthcare environment.

## 1 Introduction

Handwashing sinks and associated premise wastewater plumbing in healthcare facilities have been identified as a source of transmission for antibiotic-resistant (AR) organisms from the environment to the patient [1]. These handwashing sinks have a plumbing fixture known as a p-trap, that provide a water seal to prevent the influx of sewer gases into the living space, but also is an ideal environment for biofilm formation and may act as a reservoir to harbor these pathogens [2]. Biofilms are microbial communities, typically associated with a surface, encased in a self-produced, extracellular polymeric substance matrix (EPS) that provides structural support and defense against environmental challenges [3]. These sink and p-trap biofilms can become colonized by AR organisms [4],[5] providing them some level of protection against disinfection [6],[7] and increasing the likelihood that transmission to the patient may occur, ultimately leading to complications commonly referred to as healthcare-associated infections (HAIs). The economic impact of HAIs is conservatively in the billions of dollars (USD) and contribute to increases in morbidity and mortality [8],[9].

Carbapenem-resistant Enterobacteriaceae (CRE) are considered a serious public health threat due to decades of poor antibiotic stewardship, increasing trans-global dissemination, and a dwindling pipeline of available drugs to keep up with evolving molecular mechanisms of resistance [10]–[13]. Predictably, these organisms have been associated with colonization of the healthcare sink environment and ultimately their transmission thereafter [14],[15]. The search for novel strategies to prevent or mitigate the spread of these organisms has created newly-sparked interest in the use of bacteriophages (phages). Phages are viruses that infect bacteria; they have become increasingly valuable agents for controlling the spread of unwanted bacteria in diverse environments and applications [16]–[18]. Virulent phages, which can infect and kill their hosts in relatively short periods of time, may also produce extracellular enzymes, such as depolymerases, capable of degrading the biofilm EPS matrix; a critical component of biofilm structure and defense [19]–[21]. Also, once an infection has commenced, progeny phage released into the biofilm environment are able to repeat the infection cycle of new hosts in close proximity. Collectively, these characteristics highlight the potential that phages have as biofilm control agents.

In the present study, our goal was to formulate a phage cocktail and evaluate its ability to specifically target (*i.e.* kill) a carbapenemase-producing KPC+ *Klebsiella pneumoniae* (CPKP) strain at various stages of colonization within a multispecies drinking water biofilm community using a sink p-trap model. Many models have examined the use of phage cocktails for controlling biofilm-associated pathogens, often involving wounds [22],[23] or indwelling medical devices [24],[25]. Our approach, however, pivots towards the use of phage cocktails as an environmental application for the purpose of biocontrol of biofilm-associated AR pathogens in the healthcare environment.

## 2 Materials and methods

### 2.1. Bacteria, media, buffers, and growth conditions

Carbapenemase-producing *Klebsiella pneumoniae* strain 1016 (pKPC_UVA010) (CAV1016) containing a *bla*_KPC-2_ plasmid [26] is a blood culture isolate from the Clinical and Environmental Microbiology Branch culture collection at the Centers for Disease Control and Prevention (CDC) (Atlanta, GA). Stability of the KPC plasmid in this strain was confirmed on selected biofilm isolates throughout the study by cultivation on CHROMagar™ KPC medium (DRG International, Inc., Springfield, NJ, USA), and by the modified CIM test [27]. Bacteria were grown on trypticase soy agar (TSA) and in tryptic soy broth (TSB) during general cultivation, with some modification to culture media when indicated. Soft agar overlays used in phage enumeration had the following composition: 15 g/liter gelatin, 8 g/liter agar, 5 g/liter peptone, 3 g/liter sodium chloride, 3 g/liter beef extract, and 0.5 g/liter anhydrous manganous sulfate. Bacterial suspensions were prepared in 0.01 M (pH 7.2) phosphate buffered saline (PBS). Phage suspensions and dilutions were prepared in phage storage buffer (PSB) (5.84 g/liter NaCl, 1.06 g/liter Tris-HCl, 0.39 g/liter Tris base, 2.46 g/liter MgSO_4_·7H_2_O). Bacteria were incubated at 37 °C unless indicated otherwise; broth cultures were grown in an orbital shaker set to 200 rpm. For surfactant supplementation experiments, the non-ionic surfactant Pluronic® P103 (BASF Corporation, Ludwigshafen, Germany) was prepared by dissolving in PBS to create a stock concentration of 500 mg/L, 0.2 µm filter-sterilized and stored at room temperature. For heterotrophic plate counts (HPCs), samples were serially diluted in PBS and plated on R2A medium. For enumeration of CAV1016 from multispecies biofilms and planktonic suspensions, samples were serially diluted in PBS and plated on the selective medium m-Endo LES (Becton Dickinson, Franklin Lakes, NJ, USA).

### 2.2. Bacteriophage isolation, purification, and propagation

Bacteriophages were isolated from several liters of untreated primary influent wastewater samples collected from two separate municipal wastewater plants: Snapfinger Creek Advanced Wastewater Plant (Dekalb County, GA, USA) and RL Sutton Wastewater Reclamation Facility (Cobb County, GA, USA). Wastewater samples were allowed to settle overnight at 4 °C, followed by centrifugation (Allegra X-30R, Beckman Coulter) at 9,000 x g for 20 min to pellet particulate matter. Supernatants were filter-sterilized (0.2 µm) and stored at 4 °C. CAV1016 phages were enriched by combining 20 mL double-strength TSB, 20 mL filter-sterilized wastewater, and 0.4 mL of a log-phase culture of CAV1016 followed by incubation overnight at 37 °C at 100 rpm. Following incubation, samples were centrifuged at 4,000 x g for 30 min, filter-sterilized (0.2 µm), covered with aluminum foil and stored at 4 °C. The titer of the phage lysate was determined using the soft agar overlay (SAO) method [28]. Distinct plaque types were aseptically isolated from the SAO plates and subsequent rounds of purification were performed using the SAO method until uniform plaque formation occurred for each of the phages isolated. Subsequent propagations of phage stocks were performed in TSB using *K. pneumoniae* 1016 (CAV1016) as the host [28]. Four phage isolates, designated as follows: SNP1_2017, SNP2_2017, SNP3_2017 and RLS1_2017 were selected based on their lytic activity towards CAV1016 and depolymerase activity as determined by plaque morphology, and used for cocktail preparations. For all experiments, phage cocktails were prepared in TSB containing 10 mM CaCl_2_ and 0.5% (v/v) liquid handsoap (Kimberly-Clark, Irving, TX, USA), with the exception of surfactant experiments in which cocktails were supplemented with 0.5 mg/liter Pluronic® P103 (final concentration).

### 2.3. Assay for phage-mediated biofilm disruption

To investigate the efficacy of the phage cocktail against *K. pneumoniae*, preliminary experiments were performed on pure cultures in a 96-well plate assay. Phage-mediated biofilm disruption was quantified using a modified microtiter plate, biofilm formation assay [29]. CAV1016 biofilms were cultivated statically in flat-bottom, polystyrene 96-well microtiter plates (Corning, Corning, NY, USA) for 48 h at 37 °C in TSB containing 0.5% (v/v) liquid handsoap (Kimberly-Clark, Irving, TX, USA); designated TSB + 0.5% handsoap. Since handsoap is likely to be a component of the liquid phase in the p-trap environment, it was included as a component of the growth medium as a representative concentration that might be dispensed during a handwashing event. Spent media was aspirated and replaced with fresh TSB + 0.5% handsoap after 24 h. Following biofilm formation, wells were gently rinsed with sterile PBS (200 µl/well) three times. A phage cocktail composed of the four pre-selected phages in TSB + 0.5% handsoap + 10 mM CaCl_2_ was screened for its ability to disrupt the pre-formed biofilms. Untreated controls were formulated in the same manner without phages. Treatments were applied statically for 2 h at 37 °C at various multiplicities of infection (MOI) (0.00001–100). After treatment, contents from the wells were gently aspirated and rinsed with sterile PBS (200 µl/well) three times. Wells were stained with 0.1% (v/v) crystal violet (CV) for 15 min at 25 °C at 200 rpm. Excess CV was removed, and wells were rinsed with deionized (DI) water to remove unbound CV, followed by a period of air drying. For quantification purposes, biofilm-bound CV was dissolved with 95% ethanol (200 µL/well) and incubated at 25 °C for 15 min at 200 rpm. Absorbance at 600 nm of dissolved CV was measured using a BioTek Synergy 2 plate reader (BioTek Instruments, Winooski, VT, USA).

### 2.4. Assay for phage-mediated biocidal activity against planktonic- and biofilm-associated CAV1016

In order to simulate biofilm growth on the chrome-plated surface of a p-trap, monoculture CAV1016 biofilms were cultivated on 316L stainless steel, 1.3 cm diameter, disc coupons (BioSurface Technologies, Bozeman, MT, USA) placed in 12-well plates in TSB + 0.5% handsoap (3 mL/well) and incubated statically for 48 h at 37 °C. Biofilms were grown in triplicate for all treatment conditions tested including untreated controls. Spent media was aspirated and replaced with fresh TSB + 0.5% handsoap after 24 h. Following biofilm formation, coupons were gently rinsed three times in sterile PBS (3 mL/coupon) and transferred to a new 12-well plate for phage treatment. A four-phage cocktail supplemented with 10 mM CaCl_2_ (final concentration) and 0.5% (v/v) handsoap was applied to the biofilms at two different titers, 10^9^ PFU/mL and 10^7^ PFU/mL for 2 h at 37 °C. The cocktails were designated phage cocktail 1 (PC1) and phage cocktail 2 (PC2) respectively. The purpose of treating at two different titers was to determine if a reduction in the titer would impact the biocidal activity against biofilm-associated cells. Following treatment, coupons were gently rinsed three times in sterile PBS (3 mL/coupon) and transferred to individual, 50 mL conical tubes containing 10 mL sterile PBS. To detach biofilms from coupons, tubes were placed in a water bath sonicator (40-kHz Branson 3800, Danbury, CT, USA) and sonicated for 30 s, followed by vortexing for 30 s in a multi-tube vortexer. This step was repeated three times. Samples were serially diluted in PBS and plated on TSA for bacterial enumeration. To detect and enumerate biofilm-associated phages, 1 mL biofilm suspensions were treated with chloroform, centrifuged at 16,100 x g at 25 °C for 1 min in order to pellet cellular debris, serially diluted in phage storage buffer (PSB) followed by plating on TSA using the SAO method. Bacteria collected from the planktonic phase after treatment in the 12-well plates were enumerated by serially diluting in PBS and plating on TSA. These were designated planktonic-associated cells and represent both biofilm-dispersed and truly planktonic cells growing in the liquid phase during the treatment process. Three experimental replicates were completed.

### 2.5. Assay for determining the effects of surfactant supplementation on phage-cocktail biofilm adsorption and biocidal activity

CAV1016 biofilms were cultivated on 316L stainless steel coupons as described above. A four-phage cocktail supplemented with 10 mM CaCl_2_ (final concentration), 0.5% (v/v) handsoap and 0.5 mg/L P103 was applied to biofilms at two different titers, 10^9^ PFU/mL (PC1) and 10^7^ PFU/mL (PC2) for 2 h at 37 °C. Treatment concentrations of the surfactant were selected based on work by Donlan *et.al.* [30] indicating anti-biofilm activity by P103 in aqueous systems, as well as preliminary experiments (data not shown) screening P103 against CAV1016 specifically [31]. Coupon processing and bacterial and phage enumeration were performed as described above. Three experimental replicates were completed.

### 2.6. CDC biofilm reactor p-trap model

Biofilms composed of a drinking water microbial consortia were cultivated in CDC biofilm reactors (CBR) (Biosurface Technologies, Bozeman, MT, USA) following previously published protocols [32]–[34] with some modifications. CBRs were used to model the sink p-trap in terms of material of construction, flow dynamics, nutrient supply and microbial inoculum. Reactors contain eight coupon holders, each holding three 316 L stainless steel disc coupons for a total of 24 coupons per reactor. Once assembled, the CBRs were sterilized by autoclave at 121 °C for 30 min and subsequently filled with approximately 350 ml of autoclaved drinking water (pH 7.5, 20.0 mg/L CaCO_3_ (alkalinity), 13.6 mg/L CaCO_3_ (hardness)) (Dekalb County, Georgia). The CBRs were connected via peristaltic pump to 20 L carboys containing autoclaved drinking water, calibrated to pump at a flow rate of 16 mL/min for 25 min. At this flow rate the CBR volume was replaced each pump cycle. Pumps were connected to timers set to activate water flow at four separate times throughout the day for 7 days per week. CBRs were also connected via peristaltic pump to 1 L containers of liquid handsoap calibrated to pump at a flow rate of 2 mL/min for 1 min. Soap was pumped into the CBRs at the same intervals used for the water, but only for 5 days per week (Monday–Friday). At the beginning of each pump cycle, a mixing cycle was initiated by activating a mixing plate at a speed of 200 rpm for 30 s. CBRs were connected to a 20 L waste carboy via a discharge port. CBRs were operated at room temperature for the duration of the experiment.

The microbial consortia consisting of *Pseudomonas aeruginosa*, *Stenotrophomonas maltophilia*, *Cupriavidus metallidurans*, *Micrococcus luteus*, *Methylobacterium fujisawaense*, and *Elizabethkingia anophelis* was derived from a native biofilm consortium recovered from p-traps collected from patient rooms in an acute care hospital. Bacteria were plated on R2A agar and incubated at 25 °C for 7 days prior to inoculation to allow for adequate growth of slower-growing organisms. A day prior to CBR inoculation, each organism was transferred to tubes containing 10 mL autoclaved drinking water. Bacteria were incubated at 25 °C with shaking at 100 rpm for approximately 18 h. After this period of acclimation, the absorbance at 600 nm of each bacterial suspension was measured by spectrophotometer (Hach DR6000, Loveland, CO, USA) and adjusted to a final concentration of 10^8^ CFU/mL. The CBR inoculum was prepared by combining each bacterial suspension, in equal parts, to make a combined inoculum. The concentration of the inoculum was verified by serial dilution and plate count on R2A medium. Each CBR was inoculated with 1 mL of the combined inoculum. The CBRs were operated in batch mode for 24 h with mixing at 100 rpm to enhance microbial attachment to coupon surfaces. Following this initial batch mode, a static batch mode was initiated for 18 h before the initiation of the continuous flow phase that included interval rinses and mixing as described above.

The CBR p-trap model was operated for 28 d, allowing the microbial consortia to establish a mature biofilm. Prior to inoculating CBRs with CAV1016, reactors were sampled to determine both the density of established biofilms and to verify the absence of CAV1016 in the reactors. Biofilms were harvested aseptically from each reactor by removing a coupon holder and transferring coupons to individual 50 mL conical tubes containing 10 mL PBS. As described above, coupons were subjected to three rounds of alternating sonication and vortex cycles followed by serial dilution and plating on R2A media with incubation at 25 °C for 7 d. To confirm the absence of CAV1016 in the CBR, biofilm suspensions were also plated on the selective medium m-Endo LES and incubated at 37 °C for 24 h.

To prepare CAV1016 for inoculation into the CBR, the strain was plated on TSA and incubated overnight at 37 °C one day prior to inoculation. A 0.15 McFarland standard was prepared in 10 ml PBS, corresponding with a bacterial density of 10^8^ CFU/mL. The inoculum was serially diluted and plated to verify inoculum density. Each CBR was inoculated with 1 mL of the CAV1016 inoculum, followed by an initial batch mode with mixing at 100 rpm for 24 h. A second, static batch mode was initiated for 18 h followed by the continuous flow phase as described above. Colonization of the multispecies biofilms by CAV1016 was monitored over 21 d, with phage cocktail treatments occurring at 7, 14, and 21 d.

### 2.7. Phage cocktail treatment of drinking water biofilms colonized by CAV1016

Biofilms colonized by CAV1016 were harvested from CBRs at 7, 14, and 21 d post-inoculation as previously described with some modification. Coupon holders were aseptically removed, and coupons were removed and gently rinsed three times in sterile PBS (3 mL/coupon) and transferred to a new 12-well plate for phage treatment. A four-phage cocktail (10^9^ PFU/mL), supplemented with 10 mM CaCl_2_ (final concentration), 0.5% (v/v) handsoap, and 0.5 mg/L P103 (final concentration) was applied to biofilms and incubated statically for 2 h at both 25 °C and 37 °C. All treatments, including untreated controls were performed in triplicate. Following treatment, coupon processing and bacterial and phage enumeration were performed as described above. Four experimental replicates were completed for treatments at 37 °C and 25 °C.

### 2.8. Statistical analysis

All experiments were performed a minimum of three times. Phage-mediated biofilm disruption experiments were analyzed using one-way analysis of variance (ANOVA) (α = 0.05). Bacterial and phage counts were log_10_ transformed and differences in recovery were analyzed using two-tailed Student's *t*-tests (α = 0.05).

## 3 Results

### 3.1. Phage-mediated disruption of K. pneumoniae CAV1016 biofilms

**Figure 1.**
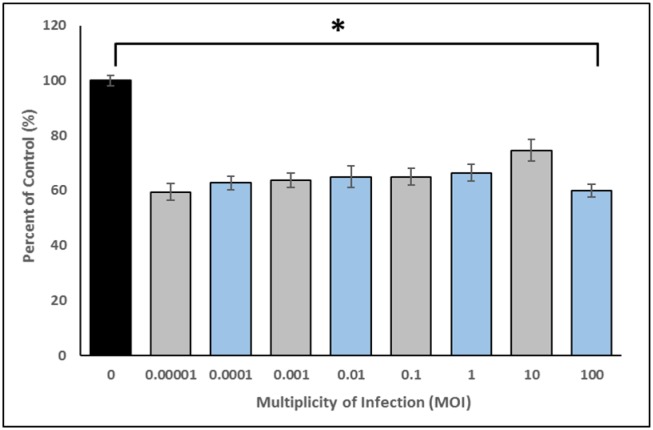
Phage-mediated disruption of 48 h *K. pneumoniae* CAV1016 biofilms. Values are means ± SEM of three replicate experiments (n = 12–24). Treatments are normalized to their corresponding untreated controls and expressed as percent of control. ANOVA indicated a significant difference between untreated controls F _(8,179)_ = 25.02, p < 0.01. *Post hoc* analysis indicated a significant difference between untreated controls and all other MOIs tested (p < 0.01). No significant difference was observed between the different MOI treatments.

Phage-mediated biofilm disruption was investigated by treating CAV1016 biofilms grown in microtiter plates, with a 4-phage cocktail for a period of 2 h at 37 °C. Both growth and treatment media were supplemented with liquid handsoap primarily to simulate conditions that would be encountered in the p-trap environment and secondly, because preliminary experiments indicated that phage-mediated disruption of the biofilm biomass was not impacted in the presence of handsoap (data not shown). The phage cocktail was applied over a wide range of MOIs (0.00001–100) in order to determine the most effective range of treatment. An average biofilm biomass reduction of approximately 35% was observed across the range of MOIs tested (Figure 1). One-way ANOVA indicated a significant difference between untreated controls and treatments. *Post hoc* analysis further indicated that all MOIs tested significantly reduced biofilm biomass relative to untreated controls; however, there were no significant differences among the MOIs tested.

### 3.2. Effect of phage treatment on planktonic- and biofilm-associated K. pneumoniae CAV1016 viability

**Figure 2.**
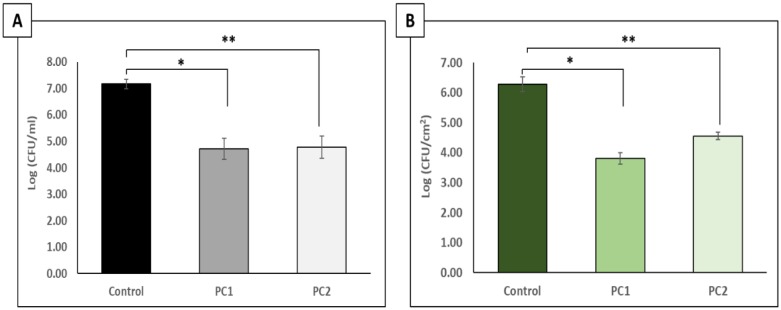
Effects of a 2 h phage cocktail treatment on *K. pneumoniae* CAV1016 grown on 316 L stainless steel coupons. A) Viable plate counts of planktonic-associated CAV1016 after 2 h phage cocktail treatments at 37 °C. Untreated controls are represented by the black bar. Phage cocktail 1(∼109 PFU/mL) (PC1, gray bar) and phage cocktail 2 (∼107 PFU/mL) (PC2, light gray bar). Values are log-normalized means ± SEM (n = 9). Single and double asterisks represent significant differences between control and the respective treatments (p < 0.05) as determined by Student's *t*-test. B) Viable plate counts of biofilm-associated CAV1016 after 2 h phage cocktail treatments at 37 °C. Untreated controls are represented by the dark green bar. Phage cocktail 1 (PC1, green bar) phage cocktail 2 (PC2, light green bar). Values are log-normalized means ± SEM (n = 9). Single and double asterisks represent significant differences between control and the respective treatments (p < 0.05) as determined by Student's *t*-test.

To determine the biocidal capacity of the phage cocktail against planktonic- and biofilm-associated CAV1016, cells were cultivated on 316 L stainless steel coupons for 48 h followed by a static, 2 h treatment at 37 °C. The phage cocktail was tested at two different titers to determine the impact that titer would have on the efficacy of the cocktail towards CAV1016 (Figure 2). The viability of CAV1016 cells dispersed from the biofilm into the planktonic phase during treatment was significantly reduced relative to untreated controls (p < 0.05) for both cocktail titers tested (Figure 2A). Reductions of approximately 2.5 log_10_ and 2.4 log_10_ CFU/mL were observed for PC1 and PC2, respectively. No significant difference was observed between PC1 and PC2 in terms of reduction in cell viability (p = 0.91). Similarly, the viability of biofilm-associated CAV1016 cells was significantly reduced after phage treatment, relative to untreated controls (p < 0.05) for both phage cocktail titers tested (Figure 2B). Reductions of approximately 2.5 log_10_ and 1.7 log_10_ CFU/mL were observed for PC1 and PC2, respectively. No significant difference was observed between PC1 and PC2 in terms of reduction in cell viability (p = 0.24).

### 3.3. Effect of surfactant supplementation on phage adsorption to biofilms and planktonic- and biofilm-associated K. pneumoniae CAV1016 viability

**Figure 3.**
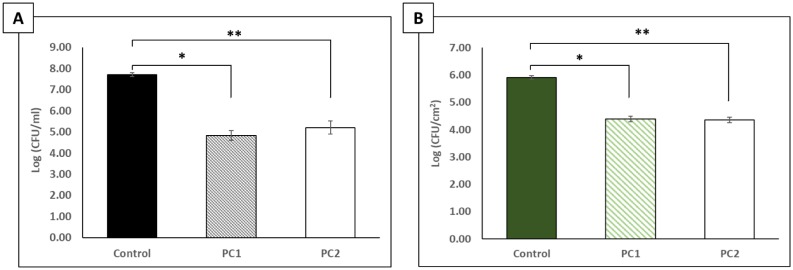
Effects of a 2 h phage cocktail treatment, supplemented with the non-ionic surfactant Pluronic® P103, on *K. pneumoniae* CAV1016 grown on 316L stainless steel coupons. A) Viable plate counts of planktonic-associated CAV1016 after 2 h, surfactant-supplemented phage cocktail treatments at 37 °C. Untreated controls are represented by the black bar. Phage cocktail 1 (∼10^9^ PFU/mL) (PC1, black diagonal bars) and phage cocktail 2 (∼10^7^ PFU/mL) (PC2, white bar). Values are log-normalized means ± SEM (n = 9). Single and double asterisks represent significant differences between control and the respective treatments (p < 0.05) as determined by Student's *t*-test. B) Viable plate counts of biofilm-associated CAV1016 after 2 h phage cocktail treatments at 37 °C. Untreated controls are represented by the dark green bar. Phage cocktail 1 (PC1, green diagonal bar) represents a high-titer treatment (∼10^9^ PFU/mL) and phage cocktail 2 (PC2, white bar) was a 1:100 dilution of PC1 (∼10^7^ PFU/mL). Values are log-normalized means ± SEM (n = 9). Single and double asterisks represent significant differences between control and the respective treatments (p <0 .05) as determined by Student's *t*-test.

To determine if supplementation of the phage cocktail with a 0.5 mg/L final concentration of a non-ionic surfactant (Pluronic® P103) could enhance the adsorption of phages to the biofilm and potentially enhance their biocidal activity, CAV1016 cells were cultivated on 316L stainless steel coupons for 48 h followed by a static, 2 h treatment at 37 °C. Preliminary experiments screening the effects of P103 (alone) against CAV1016 indicated some dispersal of biofilm biomass, but no significant effect on the viable count (data not shown) [31]. However, when the surfactant was combined with phage (surfactant-supplemented phage cocktail) and applied using the same two titers previously tested, the viable counts of CAV1016 cells dispersed from the biofilm into the planktonic phase during treatment were significantly reduced relative to untreated controls (p < 0.05) for both cocktail titers tested (Figure 3A). Reductions of approximately 2.9 log_10_ and 2.5 log_10_ CFU/mL were observed for PC1 and PC2, respectively. No significant difference was observed between PC1 and PC2 in terms of reduction in cell viable counts (p = 0.35). The viable counts of biofilm-associated CAV1016 cells were significantly reduced after phage treatment, relative to untreated controls (p < 0.05) for both phage cocktail titers tested (Figure 3B). Here, reductions of approximately 1.5 log_10_ CFU/cm^2^ were observed for both PC1 and PC2. No significant difference was observed between PC1 and PC2 in terms of reduction in cell viability (p = 0.80).

**Figure 4.**
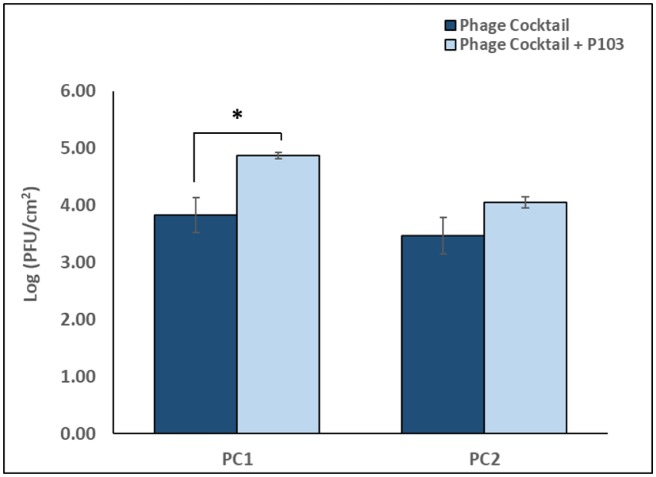
Effect of non-ionic surfactant supplementation on phage cocktail adsorption to *K. pneumoniae* CAV1016 biofilms. Navy bars represent phage cocktail 1 (PC1, ∼10^9^ PFU/mL) and phage cocktail 2 (PC2, ∼10^7^ PFU/mL) without P103. Light blue bars represent phage cocktail 1 (PC1, ∼10^9^ PFU/mL) and phage cocktail 2 (PC2, ∼10^7^ PFU/mL) with P103. Values are log-normalized means ± SEM (n = 9). Asterisk represents a significant difference (p < 0.05) between PC1 with P103 and PC1 without P103, as determined by Student's *t*-test.

Biofilm-associated phage were enumerated for phage cocktail treatments with and without P103 supplementation (Figure 4). A comparison of these treatments indicated a significant difference (p < 0.05, ∼1 log_10_ PFU/cm^2^) in the number of phages recovered from the biofilm-phase for PC1 with P103 versus PC1 without P103. When comparing PC2 with P103 versus PC2 without P103, a similar trend in recovery was observed; however, this observation was not significantly different (p = 0.09). Enumeration of phages in control biofilm samples did not indicate the presence of phages.

### 3.4. Surfactant-supplemented phage cocktail reduces both biofilm- and planktonic-associated K. pneumoniae CAV1016 viability in multispecies biofilms

**Figure 5.**
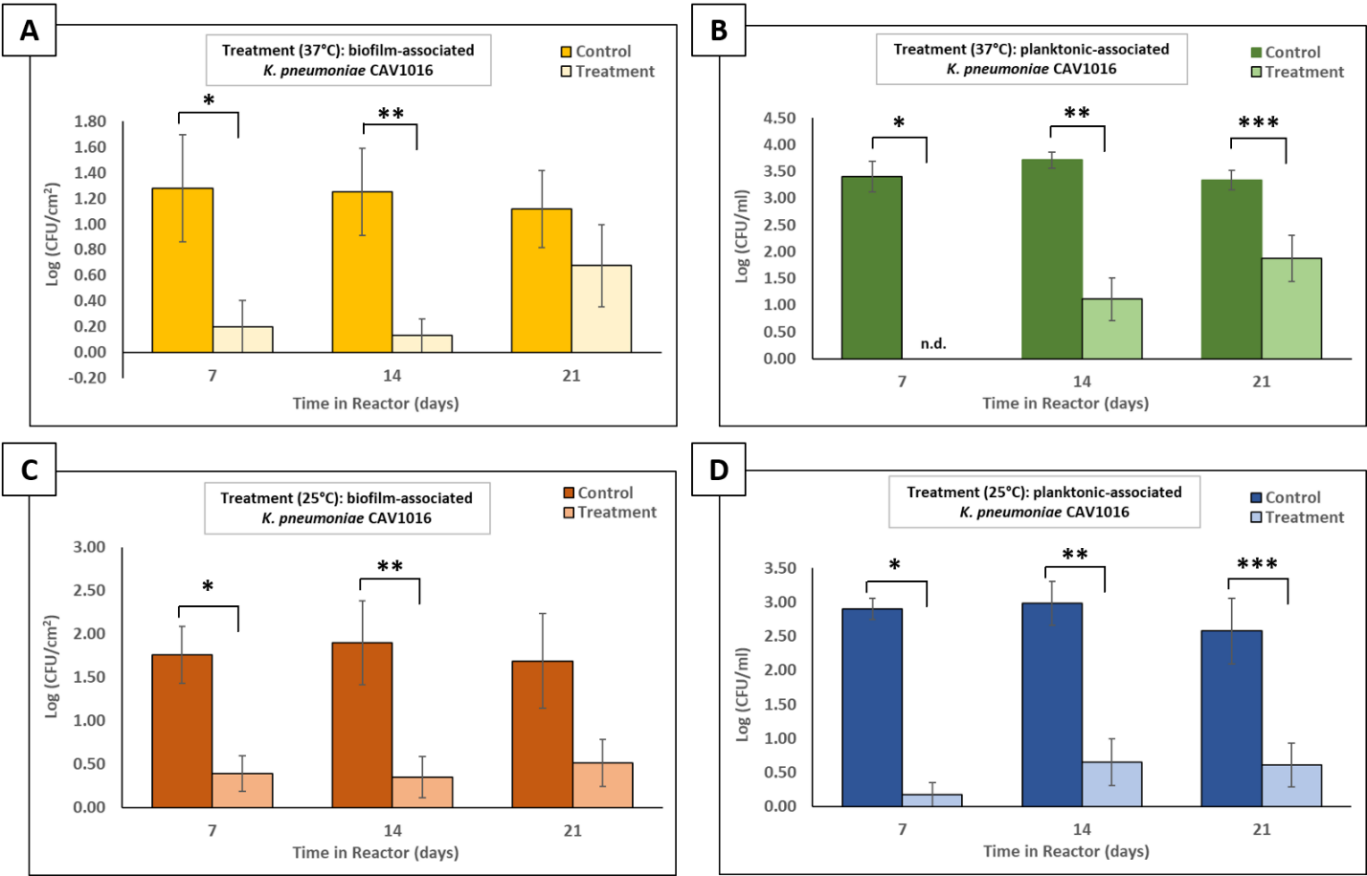
Effect of a surfactant-supplemented phage cocktail on biofilm- and planktonic-associated *K. pneumoniae* CAV1016 colonizing multispecies drinking water biofilms. A) Viable plate counts of biofilm-associated CAV1016 after 2 h, surfactant-supplemented phage cocktail treatments (10^9^ PFU/mL) at 37 °C, applied 7, 14, and 21 d post-colonization. Untreated controls are represented by gold bars and treatments are represented by light gold bars. Values are log-normalized means ± SEM (n = 12). Asterisks represent significant differences between untreated controls and their respective treatments (p < 0.05) as determined by Student's *t*-test. B) Viable plate counts of planktonic-associated CAV1016 after 2 h, surfactant-supplemented phage cocktail treatments (∼10^9^ PFU/mL) at 37 °C, applied 7, 14, and 21 d post-colonization. Untreated controls are represented by green bars and treatments are represented by light green bars. Values are log-normalized means ± SEM (n = 12). ‘N.d.’ means ‘not detected’. Asterisks represent significant differences between untreated controls and their respective treatments (p < 0.05) as determined by Student's *t*-test. C) Same as panel A but at 25 °C D) Same as panel C but at 25 °C.

A CBR p-trap model was used to test the ability of a surfactant-supplemented phage cocktail to target CAV1016 after colonizing multispecies drinking water biofilms. For treatments at 37 °C, viable counts of biofilm-associated CAV1016 were significantly reduced (p < 0.05) 7 and 14 d after colonization (Figure 5A). An approximate reduction of 1 log_10_ CFU/cm^2^, relative to untreated controls, was observed for these two time points. However, no significant difference in biofilm-associated CAV1016 viable counts was observed 21 d post-colonization as a result of treatment. Similarly, for treatments applied at 25 °C, viable counts of biofilm-associated CAV1016 were also significantly reduced (p < 0.05), 7 and 14 d after colonization (Figure 5C). Here, reductions of approximately 1.4 log_10_ and 1.6 log_10_ CFU/cm^2^ were observed for treatments at 7 and 14 d, respectively. No significant difference in biofilm-associated CAV1016 viable counts was observed 21 d post-colonization as a result of treatment.

Viable plate counts were also performed to determine the fate of CAV1016 cells dispersed from the biofilms into the planktonic-phase during treatment. For treatments at 37 °C, viable counts of planktonic-associated CAV1016 were significantly reduced (p < 0.05), relative to untreated controls, at 7, 14, and 21 d after colonization (Figure 5B). Approximate reductions of 3.4 log_10_, 2.6 log_10_, and 1.5 log_10_ CFU/mL, were observed for treatments at 7, 14, and 21 d, respectively. For treatments applied at 25 °C, viable counts of planktonic-associated CAV1016 were also significantly reduced (p < 0.05), relative to untreated control, at 7, 14, and 21 d after colonization (Figure 5D). Approximate log reductions of 2.7 log_10_, 2.3 log_10_, and 2 log_10_ CFU/mL were observed for treatments at 7, 14, and 21 d, respectively.

### 3.5. Phage cocktail treatment does not reduce the viability of biofilm- and planktonic-associated members of the microbial consortia

**Figure 6.**
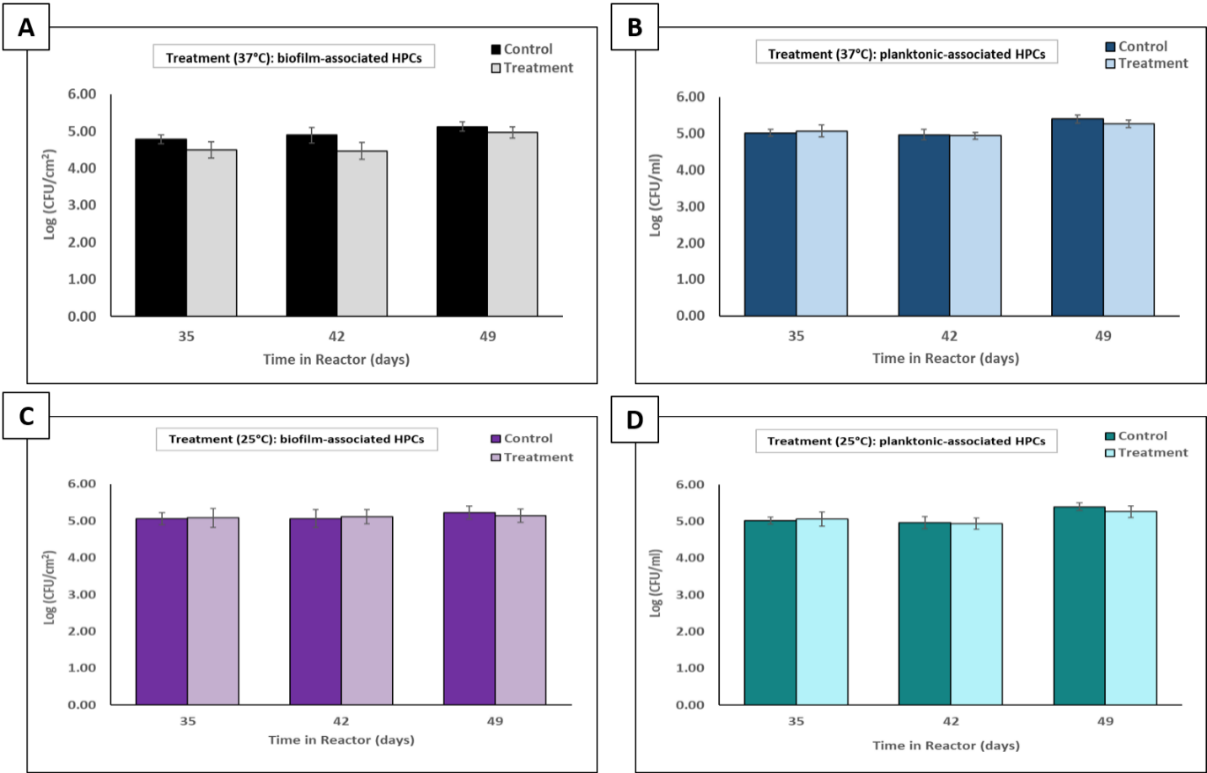
Effect of a surfactant-supplemented phage cocktail targeting *K. pneumoniae* CAV1016 on the density of biofilm- and planktonic-associated members of a microbial consortia. A) Biofilm-associated HPCs performed after 2 h, surfactant-supplemented phage cocktail treatments (10^9^ PFU/mL) at 37 °C, applied 7, 14, and 21 d post-colonization. Untreated controls are represented by black bars and treatments are represented by gray bars. Values are log-normalized means ± SEM (n = 12). No significant differences were detected between untreated controls and their respective treatments as determined by Student's *t*-test (p > 0.05). B) Planktonic-associated HPCs performed after 2 h, surfactant-supplemented phage cocktail treatments (10^9^ PFU/mL) at 37 °C, applied 7, 14, and 21 d post-colonization. Untreated controls are represented by blue bars and treatments are represented by light blue bars. Values are log-normalized means ± SEM (n = 12). No significant differences were detected between untreated controls and their respective treatments as determined by Student's *t*-test (p > 0.05). C) Same as panel A but at 25 °C. D) Same as panel B but at 25 °C.

To determine if the phage cocktail treatment had an effect on the viability of biofilm- and planktonic-associated members of the microbial consortia, heterotrophic plate counts (HPCs) were performed after each treatment. For each of the 2 h treatments applied at 37 °C, a comparison of HPCs indicated no significant difference in biofilm-associated HPCs between treated biofilms and untreated controls (p > 0.05) (Figure 6A). Similarly, for each of the treatments applied at 25 °C, no significant difference (p > 0.05) was observed in biofilm-associated HPCs between treated biofilms and untreated controls (Figure 6C). Cells dispersed from biofilms into the planktonic phase during treatment were also quantified at both 37 °C and 25 °C, with no significant difference (p > 0.05) detected between treated and untreated samples at each of the treatment intervals (Figures 6B and C).

### 3.6. Recovery of biofilm-associated phages from biofilms treated at 37 °C and 25 °C

**Figure 7.**
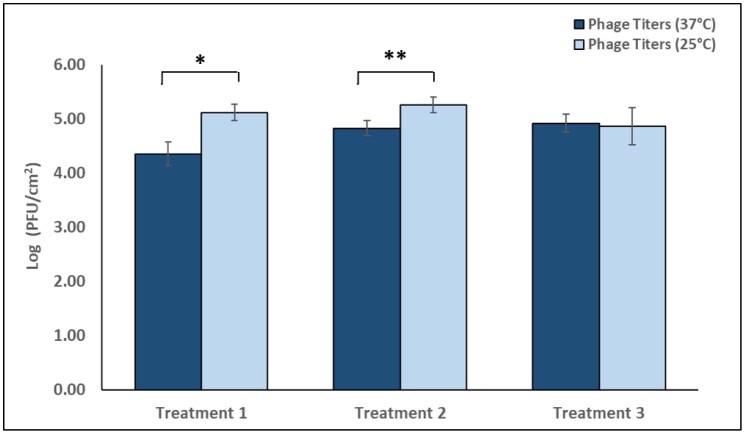
Enumeration of biofilm-associated phages recovered from drinking water biofilms colonized by *K. pneumoniae* CAV1016, after 2 h treatments at either 37 °C or 25 °C. Dark blue bars represent biofilm-associated phage titers after treatments at 37 °C. Light blue bars represent biofilm-associated phage titers after treatments at 25 °C. Treatment 1 was applied at 7 d, treatment 2 at 14 d, and treatment 3 at 21 d, post-colonization by CAV1016. All cocktails were applied at a titer of 10^9^ PFU/mL and supplemented with P103. Values are log-normalized means ± SEM (n = 12). Asterisks represents a significant difference (p < 0.05) between the number of recovered phages at 37 °C and 25 °C after a given treatment, as determined by Student's *t*-test.

The ability of phages to effectively adsorb to the biofilms at 37 °C and 25 °C was determined by enumerating biofilm-associated phages after three separate, 2 h treatments (10^9^ PFU/mL) using the SAO method (Figure 7). Treatment one, applied 7 d after CAV1016 colonization, resulted in a significantly higher recovery (p < 0.01) of phages at 25 °C (5.12 log_10_ PFU/cm^2^) versus 37 °C (4.36 log_10_ PFU/cm^2^). Similarly, treatment two, applied 14 d after CAV1016, resulted in a significantly higher recovery (p < 0.05) of phages at 25 °C (5.26 log_10_ PFU/cm^2^) versus 37 °C (4.82 log_10_ PFU/cm^2^). Treatment three, applied 21 d after CAV1016 colonization, did not result in any significant differences in the recovery of biofilm-associated phages at 25 °C (4.86 log_10_ PFU/cm^2^) or 37 °C (4.93 log_10_ PFU/cm^2^). Enumeration of phages in control biofilm samples did not indicate the presence of phages.

## 4 Discussion

Using a CBR sink p-trap model, we were able to demonstrate that a bacteriophage cocktail can target and reduce the viable count of a carbapenemase-producing KPC+ strain of *K. pneumoniae* colonizing defined, multispecies drinking water biofilms. A number of studies have investigated the role that handwashing sinks and the associated premise plumbing environment play in the transmission of AR pathogens in healthcare facilities [35],[36]. Biofilms may play a critical role in the dispersal of pathogens and AR organisms [37]. Since biofilm disruption is a desirable characteristic to have in phage cocktails geared towards targeting biofilm-associated cells, we screened for and selected phages that exhibited depolymerase activity on agar plates and subsequently tested their ability to disrupt pre-formed CAV1016 biofilms in the form of a cocktail. A wide range of MOIs were tested to identify an effective dose that could lead to biofilm disruption in a relatively short period of time (2 h). An average of 35% disruption, relative to untreated controls, was observed across all the MOIs tested with no significant difference between each treatment, suggesting that our phage cocktail could disrupt pre-formed biofilms over a wide dose range. One of the limitations of measuring biofilm disruption using a microtiter plate CV assay is that the mechanism by which disruption occurs is not clearly elucidated; specifically, if disruption is caused by cell lysis. That being said, the similarity in disruption observed over such a wide range of MOIs could be attributed to a mechanism such as the production of EPS-degrading depolymerases but could also be a result of lysis from within and lysis from without [38].

Therefore, in follow-up experiments, we cultivated CAV1016 biofilms on 316L stainless steel coupons with the goal of establishing (i) their biofilm-forming capacity on a material similar to the chrome-plated surface of hospital handwashing sink p-traps and (ii) the biocidal capacity of our phage cocktail against biofilm-associated cells. Additionally, because our previous experiments indicated similar biofilm disruption levels over a wide range of MOIs, we focused on a narrower range of titers for our cocktail treatments. CAV1016 biofilms treated for 2 h with phage cocktail titers of 10^9^ PFU/mL (PC1) and 10^7^ PFU/mL (PC2), resulted in significant reductions in viable counts relative to untreated controls (2.5 log_10_ and 1.7 log_10_ CFU/cm^2^ respectively), suggesting in part that our phage treatments had a biocidal component to their activity. The fact that treatments, 2 orders of magnitude apart resulted in similar reductions in viable counts, demonstrates the flexibility in dosing that a phage cocktail could provide due to their replicating nature. Furthermore, these findings were in agreement with our biofilm dispersal experiments that demonstrated similar levels of biofilm disruption across a wide range of MOIs. The level of biocontrol observed as a result of these treatments was extended to cells dispersed into the planktonic phase during treatment. Reductions of 2.5 log_10_ (PC1) and 2.4 log_10_ (PC2) CFU/mL, relative to untreated controls, suggest that in the presence of phages at a relatively high titer in the bulk fluid, dispersed cells are also effectively controlled. Similar observations were noted by Cornelissen *et al.* when studying the biofilm degradation properties of *Pseudomonas putida* phages [39].

Even with the use of phage cocktails, the challenge of treating biofilm-associated cells requires that multiple strategies be employed. To-that-end, we explored the use of a non-ionic surfactant (Pluronic® P103), to supplement our phage cocktail treatment. P103 is a triblock copolymer belonging to a class of water-soluble copolymers known for their versatility in applications including drug delivery as well as solubilization and stabilization of compounds [40],[41]. Surfactants (surface active agents) can alter the surface tension within biofilms and at the biofilm/substratum interface, allowing greater penetration of antimicrobial agents, and we hypothesized that P103 could provide enhanced penetration and/or adsorption of bacteriophages to the biofilm. The use of surfactants as dispersive agents, as well as for delivery of bioactive molecules is well documented [30],[42]–[44]. Treatment of CAV1016 biofilms with a surfactant-supplemented cocktail resulted in a significant reduction in viable counts (∼1.5 log_10_ CFU/cm^2^ for both PC1 and PC2) for biofilm-associated cells, relative to untreated controls. Similarly, there was also a significant reduction in the viable counts of planktonic-associated cells, relative to untreated controls (2.9 log_10_ and 2.52 CFU/mL log_10_ for PC1 and PC2 respectively). Interestingly, the number of phages recovered from biofilms after treatment with the surfactant-supplemented cocktail was significantly higher (∼1 log_10_ PFU/cm^2^) than without surfactant supplementation. This was observed for the high-titer cocktail (PC1) but not for the lower-titer cocktail (PC2). It should be noted however, that a similar trend of higher recovery was observed for PC2, but it was not statistically significant. Collectively, this indicated to us that although the biocidal activity of our phage cocktail was not enhanced by surfactant-supplementation, it did improve phage association with the biofilm, which is a key element to promoting phage-host interactions, particularly in multispecies biofilms [45].

The ability of our surfactant-supplemented phage cocktail to target CAV1016, colonizing multispecies biofilms was ultimately investigated using a CBR p-trap model. Viable counts of biofilm-associated CAV1016 were significantly reduced at both 37 °C and 25 °C during the early (7 d) and middle-stages (14 d) of colonization but not during the late-stages of colonization (21 d). This may be explained by the fact that association with the established biofilm over a longer period of time may actually act to shield cells from phage attack [46]. It also highlights the importance of early detection of suspected pathogens so that adequate control measures can be implemented [47]. Additionally, the target organism may be present in low numbers compared to other, more well-established members of the biofilm community. This could pose a challenge in reaching what has previously been described as a ‘proliferation threshold’ capable of sustaining phage populations in the biofilms [16]. The reductions in CAV1016 viable counts in both biofilm and planktonic phases did not coincide with a reduction in the viable counts of other members of the biofilm community. This observation highlights the host-specificity that phages can have towards a bacterial target within a mixed, microbial community; an observation that Cieplak and colleagues also noted when using a phage cocktail to target *Escherichia coli* associated with other commensal microbiota [48]. Notably, the present study demonstrates that reductions in viable counts of biofilm-associated CAV1016 can occur under sub-optimal growth temperatures which comes in to play when considering the application of these phages in a p-trap setting which is likely to experience fluctuations in temperature. This observation is consistent with previous studies that demonstrated that parameters such as temperature or nutrient concentrations do not eliminate susceptibility to phage infection [49]. Also, we suspect that the higher recovery of phages from biofilms treated at 25 °C with our surfactant-supplemented cocktail may be indicative of the role that a shift in P103 chemistry plays in aiding phage association with the biofilm matrix, particularly at lower temperatures [50]. Lastly, changes in the hydrodynamics of the system (*i.e.*, flow rate and shear forces) could contribute to the mechanical disruption of biofilm-associated populations [51], further contributing to their susceptibility to phage and other disinfectants

The use of phages for the purposes of biofilm control has been studied extensively over the past 25 years for a wide range of organisms and applications. For example, Roy *et.al.* studied the use of listeriaphages to target *Listeria monocytogenes* biofilms formed on stainless steel coupons and found that single or cocktail phage suspensions worked at least as well as a quaternary ammonium compound, in reducing *L.* monocytogenes populations [52]. Sillankorva and colleagues investigated the ability of phages to target dual species biofilms of *Pseudomonas fluorescens* and *Staphylococcus lentus*, concluding that phages could effectively target and reduce the population of their specific host in mixed biofilms [53]. A similar study by Chhibber *et.al.* found that mixed-species biofilms of *K. pneumoniae* and *P. aeruginosa* could be disrupted by a cocktail of phages specific to each host [54]. With many healthcare facilities facing the challenge of abating outbreaks of organisms like *Pseudomonas* and *Klebsiella*, associated with shower units and other plumbing fixtures, the use of phage cocktails could potentially be incorporated into existing disinfection and biocontrol strategies. Optimization of both the delivery mechanisms as well as the phage candidates could pave the way for improvements in the reduction of targeted biofilm-associated pathogens in a number of applications.

In many aspects, the present study confirms the findings of previous studies that investigated the use of phages for the purpose of targeting biofilms. Of course, no prospective use of phage-based biocontrol exists without some level of concern. There must be a concerted effort to address and evaluate potential risk factors to phage application such as the evolution of phage resistance in target bacterial species [55],[56] as well as the potential for horizontal gene transfer between pathogenic and non-pathogenic bacteria [57],[58]. Also, infection kinetics will vary from environment-to-environment as well as from host-to-host [59], so adequate screening must be done to ensure that the best possible phage candidates are selected for any given application. Additionally, native microbial species provide an added layer of biocontrol through their competitive ecological advantages [60]. Previous studies have shown that in the presence of autochthonous microbial communities, pathogen colonization is reduced [61]. Furthermore, phages have been shown to synergize with native microbial communities leading to effective pathogen control [62]. The use of a seven-species biofilm in our model, with the goal of targeting a single species, further advances our understanding of the capabilities that phages have in the realm of biocontrol, specifically as it pertains to environmental applications.

## 5 Conclusion

The economic and health impacts of healthcare-associated infections [8],[63] present challenges whose solutions require multi-faceted approaches. Curtailing the transmission of AR organisms from the healthcare environment to the patient is one of those critical aspects that must be addressed. In the context of handwashing sinks, targeting pathogens, specifically within biofilms, could help to alleviate the reliance on strong chemical disinfectants that can lead to damage to the built environment as well as the formation of toxic by-products [64] and further help mitigate recurring outbreaks that could lead to detrimental patient outcomes. Our work demonstrates that bacteriophage cocktails can reduce (*i.e.*, kill/control) the burden of a targeted pathogen within a multispecies drinking water biofilm community. This could provide an additional avenue of biocontrol strategies to prevent the spread of AR pathogens from the handwashing sink environment to the patient. The adequate application of phages (*i.e.*, titers and mode of delivery) onto sink surfaces and into associated plumbing fixtures (*e.g.*, p-traps), as well as environmental factors (*e.g.*, temperature) are essential to achieve the desired levels of biocontrol. Future work will primarily focus on the optimization of our phage cocktails through methods such as encapsulation as well as testing the efficacy of our phage cocktail treatments in an *in-situ* sink p-trap model.
